# Parameter estimation on multivalent ITC data sets

**DOI:** 10.1038/s41598-022-17188-x

**Published:** 2022-08-04

**Authors:** Franziska Erlekam, Maximilian Zumbansen, Marcus Weber

**Affiliations:** Zuse Institute Berlin, Takustraße 7, 14195 Berlin, Germany

**Keywords:** Applied mathematics, Biochemistry

## Abstract

The Wiseman fitting can be used to extract binding parameters from ITC data sets, such as heat of binding, number of binding sites, and the overall dissociation rate. The classical Wiseman fitting assumes a direct binding process and neglects the possibility of intermediate binding steps. In principle, it only provides thermodynamic information and not the kinetics of the process. In this article we show that a concentration dependent dissociation constant could possibly stem from intermediate binding steps. The mathematical form of this dependency can be exploited with the aid of the Robust Perron Cluster Cluster Analysis method. Our proposed extension of the Wiseman fitting rationalizes the concentration dependency, and can probably also be used to determine the kinetic parameters of intermediate binding steps of a multivalent binding process. The novelty of this paper is to assume that the binding rate varies per titration step due to the change of the ligand concentration and to use this information in the Wiseman fitting. We do not claim to produce the most accurate values of the binding parameters, we rather present a novel method of how to approach multivalent bindings from a different angle.

## Introduction

The Isothermal Titration Calorimetry (ITC) experiment, together with a method to extract binding parameters of binding interactions between a macromolecule and a ligand, was introduced by Wiseman et al. in 1989^1^. The assembly of this experiment is a reference cell containing a buffer solution, a main cell containing a fixed macromolecule concentration. Both cells are in an adiabatic jacket. The ligand concentration is titrated through a syringe into the main cell at fixed time points, evolving or absorbing heat during the binding process. In order to maintain the same temperature as in the reference cell, the main cell is heated or cooled down and the power used is being recorded. As the molar ratio between ligand and macromolecule increases with each injection, at some point saturation occurs and the heat evolved decreases. The classical ITC method would then connect the heat peaks to a sigmoidal curve. The curve slope at midpoint is $K_a$. The midpoint itself is the stoichiometry.

Based on the ITC experiment, another method was introduced in 2012 by Burnouf et al.^2^, as well as Vander Meulen and Butcher^3^, which not only extracts thermodynamic information, but also binding kinetics from the heat flow. KinITC approximates the exponential curve of every injection of the isotherm after each peak with a least squares method. Finally, the $k_{\textit{on}}$ of the binding process is determined by averaging the $k_{\textit{on}}$ rates of all the titration steps.

The method presented in this paper uses the integrated peaks of the recorded heat flow and optimizes the kinetic binding parameters such that the deviation to the actual heat flow is minimal. This method is performed for both the Wiseman fitting and the ${{Q}}_c$ fitting. Again we show that the binding rates do differ for the first and subsequent bindings and unbindings. $k_{\textit{on}}$ is ligand concentration dependent and changes thus over time.

First, the experimental binding rates for each injection can be extracted. The overall *binding rates* $k_{\textit{on}}$ and $k_{\textit{off}}$ are retrieved by calculating the average of those experimental binding rates. Their fraction defines the *dissociation constant*

$$\begin{aligned} K_d:=\frac{k_{\textit{off}}}{k_{\textit{on}}}, \end{aligned}$$

as well as the *association constant*

$$\begin{aligned} K_a:=\frac{k_{\textit{on}}}{k_{\textit{off}}}. \end{aligned}$$

Both are binding parameters extracted by the Wiseman method. The binding rates determine the binding affinity between two molecules. Having a molecule *L* and a molecule *M*, a single binding is described by

$$\begin{aligned}{}[L] + [M]\mathop {\rightleftharpoons }\limits _{k_{\textit{off}}}^{k_{\textit{on}}}[ML], \end{aligned}$$

where $[\cdot ]$ denotes the *free molecule’s concentration*.

Recently, a new method was proposed by Erlekam et al.^4^ to retrieve microscopic binding rates through the experimental binding rates extracted via kinITC. Microscopic binding rates determine the binding affinity between the binding sites of two molecules which is not constant. This is the fundamental difference to the existing kinITC method: In the classical kinITC the heat curve of each injection is used to obtain a $k_{\textit{on}}$ value which is averaged in the end to on overall $k_{\textit{on}}$ rate of the binding process. For kinITC the shapes of the heat flow peaks are essential, whereas we only need th ethermodynamic information, because these reactions are concentration dependent. The key point is that there is a concentration dependent $k_{\textit{on}}$ rate per titration step.

In^4^, the Robust Perron Cluster Algorithm (PCCA+) is used to coarse grain microscopic binding rates to modelled binding rates for each molar ratio, which are then used to fit the experimental binding rates. The transition states of complex binding processes can be modeled with row-stochastic rate matrices. PCCA+ is a spectral cluster method to reduce the dimensionality of these matrices. The result is a fuzzy clustering and each microstate gets assigned a certain membership to the two macro states: bound and unbound. In^5^ Weber and Röblitz demonstrate that this robust method always delivers a fuzzy clustering for nearly uncoupled Markov chains with transition states. The advantage compared to other clustering methods is that for PCCA+ the process must not necessarily be reversible and the matrix does not need to be decomposable.

This shown concentration dependency of the paper^4^ gave the inspiration for the following: Erlekam et al. showed an interdependance of $k_{\textit{on}}$ to the ligand concentration. In this paper, we will approach the topic of concentration dependence from another angle. In this paper we assume a dependence of the dissocation rate $k_{\textit{off}}$ and the ligand concentration.

The thermodynamic binding parameters are the enthalpy or *heat of binding* $\Delta H^{\circ }$, which defines the amount of heat absorbed or produced for a binding, the *entropy* $\Delta S^{\circ }$, which is a measure of dispersal of energy for a binding, and the *Gibbs free energy* $\Delta G^{\circ }$, the amount of available energy, and the *number of binding sites* *n*. As stated in^1^, the heat of binding, the entropy, the Gibbs free energy, and the association constant are related through the following equation

$$\begin{aligned} \Delta G^{\circ } = -RT\ln K_a = \Delta H^{\circ } - T\Delta S^{\circ }, \end{aligned}$$

where *R* is the gas constant and *T* the absolute temperature in Kelvin.

In this paper, we will compare the resulting graphs of the Wiseman fitting with the proposed ${{\mathcal{Q}}}_c$ fitting of the paper^4^ over ITC data sets. For this proof of concept we used experimental ITC data from Igde et al.^6^. The receptors used therein were lectin Concanavalin A (Con A). Depending on pH of the assay, the receptor can be bivalent or tetravalent.The ligands were glycooligomers based on oligo(amidoamines) with pendant mannose side chains. For the bivalent fitting we use bivalent Con A with the bivalent macromolecule for the bivalent fitting. For the trivalent fitting we used the trivalent Con A and the tetravalent macromolecule. Thus, the maximal compound valency depends on the minimum valency of ligand and receptor. Because we are using the Wiseman function that hold for 1:1 stoichiometries in^1^, we also assume a 1:1 stoichiometry in our examples below.

## The Wiseman fitting

Having an ITC data set of a binding between ligand *L* and macromolecule *M* with $T\in \mathbbm {N}$ injections, and the respective *sets of total concentrations* $L_t=\{l_1,\ldots ,l_T\}$ and $M_t=\{m_1,\ldots ,m_T\}$, the *Wiseman function* is defined as

$$\begin{aligned} W(K_a,n;l,m):=\frac{1}{2} \left( 1 + \frac{n-\frac{l}{m}-\frac{1}{mK_a}}{\sqrt{\left( n + \frac{l}{m} + \frac{1}{mK_a}\right) ^2 - 4n\frac{l}{m}}}\right) , \end{aligned}$$

for $l\in L_t$ and $m\in M_t$. By using the *integrated peaks of the ITC data for each injection*

$$\begin{aligned} q_{trans}:=\{(q_{trans})_1,\ldots ,(q_{trans})_T\}, \end{aligned}$$

the *Wiseman fitting* obtains the association constant, the number of binding site and the enthalpy by minimizing the difference of the hat of binding and the Wiseman function scaled with the enthalpy. For this minimization the Frobenius norm is used:

1 $$\begin{aligned} \min _{K_a,n,\Delta H^{\circ }}\big \Vert q_{trans} - W(K_a,n;L_t,M_t)\Delta H^{\circ }\big \Vert . \end{aligned}$$

Note, that in this expression the term *W* is without physical unit. Thus, the unit of $q_{trans}$ will correspond to the physical unit of $\Delta H^{\circ }$. Scaling $q_{trans}$ just scales $\Delta H^{\circ }$. In order to be without physical unit, $K_a$ has to reverse the physical unit of *m*. A common rescaling of *m* and *l* only changes the physical unit of $K_a$.

## The ${\mathcal{Q}}_c$ fitting

As the exisitng methods^2,4^ suggest, it is valid for multivalent data sets to assume a connection between molar ratio and association or dissociation. With this in mind, a new fitting can be specified by using *microscopic binding rates* for an *s*-valent binding

$$\begin{aligned} k_{\textit{on}_{1}}\text { and }k_{\textit{off}_{1}}&\text { for the first microscopic binding,}\\ k_{\textit{on}_{2}}\text { and }k_{\textit{off}_{2}}&\text { for the second consecutive microscopic binding,}\\&\vdots \\ k_{\textit{on}_{s}}\text { and }k_{\textit{off}_{s}}&\text { for the s-th consecutive microscopic binding,} \end{aligned}$$

for $s\in \mathbbm {N}$. To retrieve association constants for each molar ratio, the PCCA+ algorithm from Weber^5^ is used to coarse grain a transition rate matrix of the microscopic binding rates *Q* to a matrix $Q_{c}$ containing the (macroscopic) binding rates. The details of how to obtain a dimension reduction via coarse graining is described in^7^. It is important to mention here, that this type of analysis just provides the relative ratios of the rates, but can not figure out the absolute physical unit of them. Thus, providing the $k_{on}$ and $k_{off}$ values with physical units does not make sense here. Following the method of^4^, the bivalent transition rate matrix is

2 $$\begin{aligned} {{\mathcal{Q}}}^T = \begin{pmatrix} -4\alpha &{} k_{\textit{off}_{1}} &{} k_{\textit{off}_{1}} &{} k_{\textit{off}_{1}} &{} k_{\textit{off}_{1}} &{} 0 &{} 0 \\ \alpha &{} -\beta &{} 0 &{} 0 &{} 0 &{} k_{\textit{off}_{2}} &{} 0 \\ \alpha &{} 0 &{} -\beta &{} 0 &{} 0 &{} 0 &{} k_{\textit{off}_{2}} \\ \alpha &{} 0 &{} 0 &{} -\beta &{} 0 &{} k_{\textit{off}_{2}} &{} 0 \\ \alpha &{} 0 &{} 0 &{} 0 &{} -\beta &{} 0 &{} k_{\textit{off}_{2}} \\ 0 &{} k_{\textit{on}_{2}} &{} 0 &{} k_{\textit{on}_{2}} &{} 0 &{} -2k_{\textit{off}_{2}} &{} 0 \\ 0 &{} 0 &{} k_{\textit{on}_{2}} &{} 0 &{} k_{\textit{on}_{2}} &{} 0 &{} -2k_{\textit{off}_{2}} \end{pmatrix}, \end{aligned}$$

with $\alpha := k_{\textit{on}_{1}}[L],\beta := -k_{\textit{off}_{1}}-k_{\textit{on}_{2}}$. For the trivalent case, see the Supplementary File. To get from the transposed transition rate matrix ${{\mathcal{Q}}}^T$ to its coarse grained transposed counterpart ${{\mathcal{Q}}}^T_c$, the PCCA+ algorithm introduces

$$\begin{aligned} {{\mathcal{Q}}}_c^T = (\chi ^T \Pi \chi )^{-1}\chi ^T \Pi {{\mathcal{Q}}}^T\chi , \end{aligned}$$

where $\Pi := \mathrm {diag}(\pi )$ with stationary distribution $\pi$ of the state space *S* related to ${{\mathcal{Q}}}$, and fuzzy membership matrix $\chi$. To retrieve $\chi$, the inner simplex algorithm from^8^ is used. By using the interpretation of Weber^9^, the coarse grained matrix yields

$$\begin{aligned} {{\mathcal{Q}}}_c^T = \begin{pmatrix} -k_{\textit{on}}[L] &{} k_{\textit{off}}\\ k_{\textit{on}}[L] &{} -k_{\textit{off}}\end{pmatrix}, \end{aligned}$$

from which $K_a$ can be retrieved.

The transition rate matrix ${Q}$ requires the ligand conctration [*L*], which will be calculated iteratively for each injection *i* as

$$\begin{aligned} {[}L]_i = {\left\{ \begin{array}{ll} l_1, &{}\text {if} i=1,\\ {[}L]_{i-1} + l_{i} - (L_{b})_{i-1}, &{}\text {otherwise,} \end{array}\right. } \end{aligned}$$

where

$$\begin{aligned} (L_{b})_{i} := \frac{n m_{i}+l_{i}+\frac{1}{K_a} - \sqrt{\left( nm_{i}+l_{i}+\frac{1}{K_{a}}\right) ^{2} - 4nm_{i}l_{i}}}{2} \end{aligned}$$

is the *bound ligand concentration for injection i*. For an *s*-valent binding, we denote

$$\begin{aligned} {{Q}}(k_{\textit{on}_{1}},k_{\textit{off}_{1}},\ldots ,k_{\textit{on}_{s}},k_{\textit{off}_{s}};[L])={K_1,\ldots ,K_T}, \end{aligned}$$

with the set of free ligand concentrations [*L*] and, on the right side, the resulting association constants $K_i$ for each injection $i\in \{1,\ldots ,T\}$. With the Frobenius norm, we then define the ${{\mathcal{Q}}}_c$ fitting as

3 $$\begin{aligned} \min _{\begin{array}{c} k_{\textit{on}_{1}},\ldots ,k_{\textit{on}_{s}}\\ k_{\textit{off}_{1}},\ldots ,k_{\textit{off}_{s}} \\ n,\Delta H^{\circ } \end{array}}\big \Vert q_{trans} - W({{Q}}(\ldots ;[L]),n;L_t,M_t)\Delta H^{\circ }\big \Vert . \end{aligned}$$

## Extracting the experimental data

To extract the total concentrations $L_t$ and $M_t$ from the ITC experiments, we follow the supporting information’s appendix I of Egawa et al.^10^, where

$$\begin{aligned} l_i&= c_L \left( \sum _{k=1}^{i}V_k\right) \frac{2V_0 - \sum _{k=1}^{i}V_k}{2(V_0)^2}&\\ m_i&= c_M \frac{2V_0 - \sum _{k=1}^{i}V_k}{2V_0 + \sum _{k=1}^{i}V_k},&\end{aligned}$$

with main cell volume $V_0$, containing the macromolecules, successive injected volumes $V_i,~i\in \{1,\ldots ,T\}$, containing the ligands, and concentrations of ligand $c_L$ and of macromolecule $c_M$. Thus, the total volume is $V_{Tot}=V_0 + \sum _{i=1}^{T}V_i$.

For the bivalent case, the values are

$$\begin{aligned} c_M&=0.071\mathrm{mmol/l}&V_0&=1.442\mathrm{ml}&\\ c_L&=0.7\mathrm{mmol/l}&V_1&=0.001\mathrm{ml},\qquad V_j=0.01\mathrm{ml},&\end{aligned}$$

and for the trivalent case

$$\begin{aligned} c_M&=0.0918\mathrm{mmol/l}&V_0&=1.442\mathrm{ml}&\\ c_L&=0.978\mathrm{mmol/l}&V_1&=0.001\mathrm{ml},\qquad V_j=0.01\mathrm{ml},&\end{aligned}$$

with $j\in \{2,\ldots ,14\}$. In the following, we will compare the quality between the Wiseman fitting and the proposed ${{\mathcal{Q}}}_c$ fitting.

## Comparison of fittings

For the Wiseman fitting, the peaks of the heat curve are integrated with univariate splines of degree 5. The parameters $K_a$, *n* and $\Delta H^{\circ }$ are obtained by a Nelder-Mead minimization.

For the ${{\mathcal{Q}}}_c$ fitting, the algorithm is implemented as a random search loop with breaking condition when a better norm, compared to the Wiseman fitting, is found. When a suitable norm threshold is reached, a Nelder-Mead minimization is applied on the ${{\mathcal{Q}}}_c$ fitting over parameters *n* and $\Delta H^{\circ }$ to find the optimal solution to the current microscopic binding rates.

In the following subsection we present the Wiseman fitting to experimental data from Igde et al.^6^. In this work, the authors selected data sets of bi- and trivalent ligands binding to tetrameric Con A with four binding sites from a series of measurements. The precision glycomacromolecule were mannose ligands with oligomer scaffolds. Since we assume a 1:1 stoichiometry, the binding process can only have the valency of the respective ligand, no matter if the receptor has more potential binding sites.

### Bivalent ligand case

**Figure 1:**
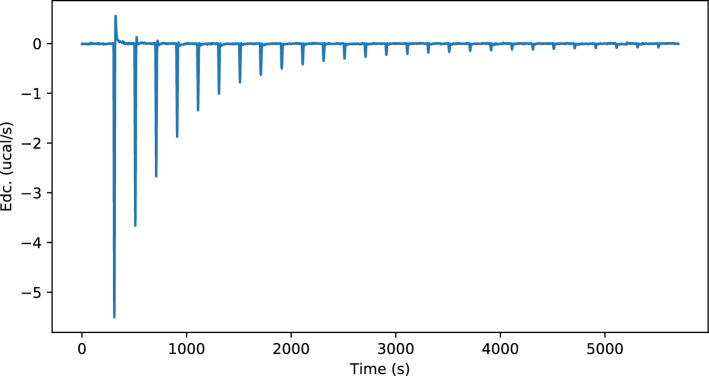
Plot of the deconvolved power trace (Edc) of bindings between a decavalent Con A and a bivalent precision glycomacromolecule over time and through 14 injections.

The bivalent case, where Con A and a bivalent precision glycomacromolecules bind together, resolves into the ITC plot as depicted in Fig. 1:

The heat evolved is extracted by integration over the 14 injection peaks. The Wiseman fitting returns the following binding parameters

$$\begin{aligned} K_a&=93.3295&n&=0.1998&\\ \Delta H^{\circ }&=-132.3069\mathrm{kcal/mol}&\end{aligned}$$

and results in an error of 5.4838.

For the ${{\mathcal{Q}}}_c$ fitting, the retrieved binding parameters are

$$\begin{aligned} k_{\textit{on}_{1}}&=136.4185&k_{\textit{off}_{1}}&=2.3716&\\ k_{\textit{on}_{2}}&=33.4088&k_{\textit{off}_{2}}&=18.5925&\\ n&=0.3208&\Delta H^{\circ }&=-73.4629\mathrm{kcal/mol}&. \end{aligned}$$

**Figure 2:**
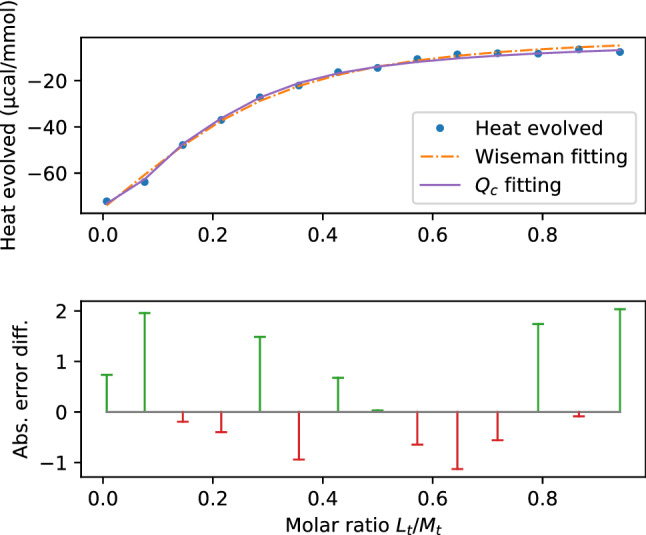
(**a**) Integrated heat per injection over molar ratio with Wiseman graphs of Wiseman and ${{\mathcal{Q}}}_c$ fitting for the bivalent case. (**b**) Difference of absolute error plots from Wiseman and ${{\mathcal{Q}}}_c$ fitting.

The error for this fitting is 3.435. The results of the ${Q}_C$ and the Wiseman fitting is shown in Fig. 2(a). Their respective errors are shown in Fig. 2(b).

When comparing the binding parameters, the heat of binding $\Delta H^{\circ }$ from the Wiseman fitting is almost double the value from the ${{\mathcal{Q}}}_c$ fitting, whereas the number of sites *n* is only two thirds.

The expected value for *n* is 0.2, which is better represented through the Wiseman fitting.

The association constant $K_a$ is not comparable to the microscopic association constants

$$\begin{aligned} K_1&:={k_{\textit{on}_{1}}}/{k_{\textit{off}_{1}}}=57.5217&\\ K_2&:={k_{\textit{on}_{2}}}/{k_{\textit{off}_{2}}}=1.7969.&\end{aligned}$$

The latter signal a stronger first and a very weak second binding. As has been said before: Because of the lack of physical units, the analysis just allows for this “relative”, qualitative result.

### Trivalent ligand case

**Figure 3:**
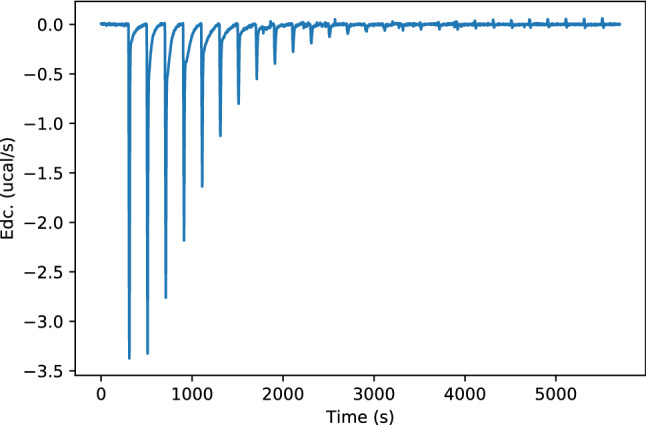
Plot of the deconvolved power trace (Edc) of bindings between a trivalent Con A and a tetravalent precision glycomacromolecule over time.

We will now examine the ITC plot resulting from a binding between a trivalent Con A and a tetravalent Precision Glycomacromolecule, as depicted in Fig. 3.

Compared to the bivalent case, the heat curve resolves in a distinct slope at molar ratio 0.3. As the slope is the most crucial part when determining the binding constants, before using the Frobenius norm, the absolute difference between fitting and $q_{trans}$ is multiplied by a Gaussian curve with mean $\mu =0.3$ and standard deviation $\sigma =0.1758$. The first injection is considered an outlier and is excluded from the minimization.

For the Wiseman fitting, the resulting binding parameters are

$$\begin{aligned} K_a&=410.2917&n&=0.3586&\\ \Delta H^{\circ }&=-127.7664\mathrm{kcal/mol},&\end{aligned}$$

and the error is 13.1455.

**Figure 4:**
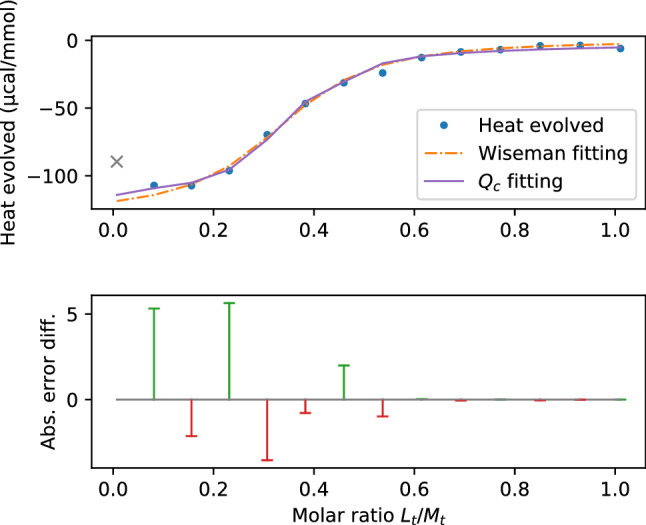
(**a**) Integrated heat per injection over molar ratio with Wiseman graphs of Wiseman and ${{\mathcal{Q}}}_c$ fitting for the trivalent case. (**b**) Difference of absolute error plots from Wiseman and ${{\mathcal{Q}}}_c$ fitting. Calculated with a Gaussian distribution to apply weight around the slope.

The ${{\mathcal{Q}}}_c$ fitting results in binding parameters

$$\begin{aligned} k_{\textit{on}_{1}}&=559.1484&k_{\textit{off}_{1}}&=1844.5886&\\ k_{\textit{on}_{2}}&=489.7665&k_{\textit{off}_{2}}&=81.966&\\ k_{\textit{on}_{3}}&=1056.7834&k_{\textit{off}_{3}}&=1338.633&\\ n&=0.3624 \quad \Delta H^{\circ }=-115.1639\mathrm{kcal/mol} \end{aligned}$$

with an error of 11.1979. The results of the  $Q_C$ and the Wiseman fitting are shown in Fig. 4(a). Their respective errors are shown in Fig. 4(b).

This time the number of sites *n* is almost identical for both fittings and the heat of binding is fairly equal.

For the microscopic association constants, we have

$$\begin{aligned} K_1&=0.3031&K_2&=5.9752&K_3&=0.7894,&\end{aligned}$$

which are very low numbers compared to $K_a$ from the Wiseman fitting. The second binding appears to be the easiest, whereas the first and the last are less frequent.

## Model selection

There is not much freedom of determining the correct model for the interpretation of the experimental data. The Wiseman fitting itself is assumed to be the fixed root model for the interpretation of the ITC data in terms of association and dissociation constants. The model ${\mathcal{Q}}$ for the detailed kinetic process depends on the chemical nature of the substances. The elementary binding events (the stoichiometry) only depend on the number of binding sites and ligand sites

The only free choice we made is to project the matrix ${\mathcal{Q}}$ to a two-dimensional matrix ${\mathcal{Q}}_c$, because we assume that the only dominating process is the “slow” overall binding event, which we hope to model correctly and robustly by the coarse grained matrix.

The choice of the projection space has been discussed in terms of “implied timescales”. It has been analyzed in terms of the separation of the timescale of the projected process with regard to the next faster process11. The first eigenvalue $\lambda_1$ of the ${\mathcal{Q}}_c$-matrix is always 0, thus this separation of timescales can be estimated from taking the ratio of the second and third eigenvalue of ${\mathcal{Q}}_c$, $\frac{{\lambda _{2} }}{{\lambda _{3} }}$. In case of the bivalent example, this ratio grows from 1.1 in the first titration step to 2 in the last titration step, which means that the second faster process is already two times faster than the slowest one (at the end of the ITC experiment). This indicates a good separation and choice of modelling. In the case of the trivalent example, however, this ratio is always at about 1.14 and indicates a bad separation.

If we would model ${\mathcal{Q}}_c$ as a two-step binding process instead of a one-step binding, then the timescales separation would be given by the ratio of the third and forth eigenvalues of ${\mathcal{Q}}$, i.e. $\frac{{\lambda _{4} }}{{\lambda _{3} }}$. In this case this ratio grows from 1.0 in the first titration step to 4.7 in the last one along the ITC experiment. However, this would not allow for a comparison with regard to the applied Wiseman fitting.

## Error Estimates

To test the robustness of this fitting, the ITC input data was disturbed by up to 1%, i.e. every value was multiplied by a random variable $x_{i} \in \left[ {0.99,1.01} \right]$.

In the bivalent example we have the following changes after disturbing the input: n is 0.33% higher, ΔH decreased by 0.31% and the best norm decreased by 0.18% compared to the undisturbed model. The k_on1_ , k_on2_ , k_off1_ and k_off2_ differ only by a factor of 10^−6^ from the disturbed model binding parameters. Therefore, K_1_ and K_2_ do not differ either. Thus, we have a stable model for the bivalent case.

The trivalent case is more complicated. The binding parameters of the undisturbed and undisturbed models differ up to 743%. K_1_, K_2_ and K_3_ therefore also differ substantially, up to 653%. However, with the different optimum found in the disturbed setting, the stoichiometry and the heat of binding are similar. In the disturbed example, n is only 0.04% higher, ΔH decreased by 1.5% and the best norm decreased by 1.36% compared to the undisturbed model. To summarize, for the trivalent binding, the ${\mathcal{Q}}_c$ fitting may not deliver satisfying results. This may be due to the same reason that we discovered in the spectral gap analysis. Possibly, the Wiseman fitting is not suitable for trivalent bindings, because there are actually three macro states instead of two. For the ${\mathcal{Q}}_c$ fitting, this is an interesting insight, because for trivalent bindings we would need to project onto a 3×3 clustered rate matrix instead of a 2×2 matrix.

## Conclusion

In this paper we have shown the ${{Q}}_c$ and the Wiseman fitting for two numerical examples. We conclude that with PCCA+ the fitting of binding parameters of bivalent and trivalent ligands is better in terms of absolute errors than the Wiseman fitting. We demonstrated the results of particular association constants for each subsequent binding step. The major advantage of the proposed method is to gain kinetic information of each binding step rather than for the whole binding process only. However, the binding parameters are just “qualitative” not quantitative. Whereas, kinITC assumes concentration independent binding associations. Our method makes use of the systematic change of the association “constants” along the measurements.

## Outlook

By taking microscopic binding and concentrations into account, we have proven the existence of a better fitting of bivalent and trivalent ITC data sets. However, the current algorithm for the ${{\mathcal{Q}}}_c$ fitting utilizes random search to find the binding parameters. An algorithm for an optimal solution is currently not available and remains a problem for future research.

As the resulting fitting relies on a transition matrix and the interpretation of its coarse grained counterpart, the approach of this paper could contribute to a model which is able to explain even more complex heat curves, where the Wiseman graph is no longer satisfactory.

Our paper only shows a proof of concept for a bivalent and a trivalent model. In the future the algorithm can be tested for bindings of higher valencies to learn more about its limitations.

Further, we have assumed a 1:1 stoichiometry that does not necessarily occur in all molecular binding experiments. The theory could therefore be generalized for other stoichiometries as a future piece of research.

Lastly, as a goodness of fit metric we used the fitting error. The two models could be further compared using different criteria, such as the Bayesian information criterion. Apart from that, a parameter study can be of interest to show which of the binding parameters are correlated.

## Supplementary Information

Supplementary Information.

## Data Availability

The data and the algorithm that support the findings of this study are openly available at https://github.com/mazumba/si-multivalent-params.
