# Supplementary material for: Parameter estimation on multivalent ITC data sets
Source: Sci Rep. 2022 Aug 4;12:13402. doi: 10.1038/s41598-022-17188-x (PMC9352788; doi:10.1038/s41598-022-17188-x)
Supplement: Supplementary file 1 — Supplementary Information. [file 41598_2022_17188_MOESM1_ESM.pdf]

Supplementary Information

In this Supplementary Information to the paper 'Parameter estimation on multivalent ITC data sets' by Zumbansen, Erlekam the rate matrix for the trivalent binding process is presented.
All computations for both bivlaent and trivalent bindings presented in the paper were conducted in Python 3.7.

The Q^T for trivalent bindings

The transposed transition rate matrix for a bivalent binding was already introduced in (2) on page 2. The transposed transition rate matrix for a trivalent binding is

$$\mathbb{R}^{22\times 22} \ni \boldsymbol{Q}^T = \begin{pmatrix} A & B & C & D \end{pmatrix},$$

with

$$A := \begin{pmatrix}
-9k_{on_1} & k_{off_1} & k_{off_1} & k_{off_1} & k_{off_1} \\
k_{on_1} & -\alpha & 0 & 0 & 0 \\
k_{on_1} & 0 & -\alpha & 0 & 0 \\
k_{on_1} & 0 & 0 & -\alpha & 0 \\
k_{on_1} & 0 & 0 & 0 & -\alpha \\
k_{on_1} & 0 & 0 & 0 & 0 \\
k_{on_1} & 0 & 0 & 0 & 0 \\
k_{on_1} & 0 & 0 & 0 & 0 \\
k_{on_1} & 0 & 0 & 0 & 0 \\
k_{on_1} & 0 & 0 & 0 & 0 \\
0 & k_{on_2} & 0 & 0 & 0 \\
0 & 0 & 0 & k_{on_2} & k_{on_2} \\
0 & 0 & k_{on_2} & 0 & 0 \\
0 & 0 & k_{on_2} & 0 & 0 \\
0 & k_{on_2} & 0 & 0 & 0 \\
0 & 0 & 0 & k_{on_2} & 0 \\
0 & 0 & 0 & 0 & k_{on_2} \\
0 & 0 & 0 & 0 & 0 \\
0 & 0 & 0 & 0 & 0 \\
0 & 0 & 0 & 0 & 0 \\
0 & 0 & 0 & 0 & 0 \\
0 & 0 & 0 & 0 & 0
\end{pmatrix},$$

$$B := \begin{pmatrix}
k_{off_1} & k_{off_1} & k_{off_1} & k_{off_1} & k_{off_1} \\
0 & 0 & 0 & 0 & 0 \\
0 & 0 & 0 & 0 & 0 \\
0 & 0 & 0 & 0 & 0 \\
0 & 0 & 0 & 0 & 0 \\
-\alpha & 0 & 0 & 0 & 0 \\
0 & -\alpha & 0 & 0 & 0 \\
0 & 0 & -\alpha & 0 & 0 \\
0 & 0 & 0 & -\alpha & 0 \\
0 & 0 & 0 & 0 & -\alpha \\
k_{on_2} & 0 & 0 & 0 & 0 \\
0 & 0 & 0 & 0 & 0 \\
0 & k_{on_2} & 0 & 0 & 0 \\
0 & 0 & k_{on_2} & 0 & 0 \\
0 & 0 & 0 & 0 & k_{on_2} \\
0 & 0 & 0 & k_{on_2} & 0 \\
0 & 0 & 0 & k_{on_2} & 0 \\
k_{on_2} & 0 & 0 & 0 & k_{on_2} \\
0 & k_{on_2} & k_{on_2} & 0 & 0 \\
0 & 0 & 0 & 0 & 0 \\
0 & 0 & 0 & 0 & 0 \\
0 & 0 & 0 & 0 & 0
\end{pmatrix}$$

where $\alpha := k_{off_1} + 2k_{on_2}$. And

$$C := \begin{pmatrix}
0 & 0 & 0 & 0 & 0 & 0 \\
k_{off_2} & 0 & 0 & 0 & k_{off_2} & 0 \\
0 & 0 & k_{off_2} & k_{off_2} & 0 & 0 \\
0 & k_{off_2} & 0 & 0 & 0 & k_{off_2} \\
0 & k_{off_2} & 0 & 0 & 0 & 0 \\
k_{off_2} & 0 & 0 & 0 & 0 & 0 \\
0 & 0 & k_{off_2} & 0 & 0 & 0 \\
0 & 0 & 0 & k_{off_2} & 0 & 0 \\
0 & 0 & 0 & 0 & k_{off_2} & 0 \\
0 & 0 & 0 & 0 & 0 & k_{off_2} \\
-\beta & 0 & 0 & 0 & 0 & 0 \\
0 & -\beta & 0 & 0 & 0 & 0 \\
0 & 0 & -\beta & 0 & 0 & 0 \\
0 & 0 & 0 & -\beta & 0 & 0 \\
0 & 0 & 0 & 0 & -\beta & 0 \\
0 & 0 & 0 & 0 & 0 & -\beta \\
0 & 0 & 0 & 0 & 0 & 0 \\
0 & 0 & 0 & 0 & 0 & 0 \\
0 & 0 & 0 & 0 & 0 & 0 \\
k_{on_3} & 0 & 0 & 0 & k_{on_3} & 0 \\
0 & 0 & k_{on_3} & k_{on_3} & 0 & 0 \\
0 & k_{on_3} & 0 & 0 & 0 & k_{on_3}
\end{pmatrix}$$

$$D := \begin{pmatrix}
0 & 0 & 0 & 0 & 0 & 0 \\
0 & 0 & 0 & 0 & 0 & 0 \\
0 & 0 & 0 & 0 & 0 & 0 \\
0 & 0 & 0 & 0 & 0 & 0 \\
k_{off_2} & 0 & 0 & 0 & 0 & 0 \\
0 & 0 & k_{off_2} & 0 & 0 & 0 \\
0 & k_{off_2} & 0 & 0 & 0 & 0 \\
0 & k_{off_2} & 0 & 0 & 0 & 0 \\
0 & 0 & k_{off_2} & 0 & 0 & 0 \\
k_{off_2} & 0 & 0 & 0 & 0 & 0 \\
0 & 0 & 0 & k_{off_3} & 0 & 0 \\
0 & 0 & 0 & 0 & 0 & k_{off_3} \\
0 & 0 & 0 & 0 & k_{off_3} & 0 \\
0 & 0 & 0 & 0 & k_{off_3} & 0 \\
0 & 0 & 0 & k_{off_3} & 0 & 0 \\
0 & 0 & 0 & 0 & 0 & k_{off_3} \\
-\beta & 0 & 0 & 0 & 0 & k_{off_3} \\
0 & -\beta & 0 & k_{off_3} & 0 & 0 \\
0 & 0 & -\beta & 0 & k_{off_3} & 0 \\
0 & k_{on_3} & 0 & -3\delta & 0 & 0 \\
0 & 0 & k_{on_3} & 0 & -3\delta & 0 \\
k_{on_3} & 0 & 0 & 0 & 0 & -3\delta
\end{pmatrix},$$

where $\beta := k_{on_3} + 2k_{off_2}$ and $\delta := k_{off_3}$.
